# DockCoV2: a drug database against SARS-CoV-2

**DOI:** 10.1093/nar/gkaa861

**Published:** 2020-10-09

**Authors:** Ting-Fu Chen, Yu-Chuan Chang, Yi Hsiao, Ko-Han Lee, Yu-Chun Hsiao, Yu-Hsiang Lin, Yi-Chin Ethan Tu, Hsuan-Cheng Huang, Chien-Yu Chen, Hsueh-Fen Juan

**Affiliations:** Taiwan AI Labs, Taipei 10351, Taiwan; Taiwan AI Labs, Taipei 10351, Taiwan; Taiwan AI Labs, Taipei 10351, Taiwan; Taiwan AI Labs, Taipei 10351, Taiwan; Taiwan AI Labs, Taipei 10351, Taiwan; Taiwan AI Labs, Taipei 10351, Taiwan; Taiwan AI Labs, Taipei 10351, Taiwan; Institute of Biomedical Informatics, National Yang-Ming University, Taipei 11221, Taiwan; Taiwan AI Labs, Taipei 10351, Taiwan; Department of Biomechatronics Engineering, National Taiwan University, Taipei 10617, Taiwan; Taiwan AI Labs, Taipei 10351, Taiwan; Graduate Institute of Biomedical Electronics and Bioinformatics, National Taiwan University, Taipei 10617, Taiwan; Department of Life Science, National Taiwan University, Taipei 10617, Taiwan

## Abstract

The current state of the COVID-19 pandemic is a global health crisis. To fight the novel coronavirus, one of the best-known ways is to block enzymes essential for virus replication. Currently, we know that the SARS-CoV-2 virus encodes about 29 proteins such as spike protein, 3C-like protease (3CLpro), RNA-dependent RNA polymerase (RdRp), Papain-like protease (PLpro), and nucleocapsid (N) protein. SARS-CoV-2 uses human angiotensin-converting enzyme 2 (ACE2) for viral entry and transmembrane serine protease family member II (TMPRSS2) for spike protein priming. Thus in order to speed up the discovery of potential drugs, we develop DockCoV2, a drug database for SARS-CoV-2. DockCoV2 focuses on predicting the binding affinity of FDA-approved and Taiwan National Health Insurance (NHI) drugs with the seven proteins mentioned above. This database contains a total of 3,109 drugs. DockCoV2 is easy to use and search against, is well cross-linked to external databases, and provides the state-of-the-art prediction results in one site. Users can download their drug-protein docking data of interest and examine additional drug-related information on DockCoV2. Furthermore, DockCoV2 provides experimental information to help users understand which drugs have already been reported to be effective against MERS or SARS-CoV. DockCoV2 is available at https://covirus.cc/drugs/.

## INTRODUCTION

From December 2019 to June 2020, SARS-CoV-2 has infected over 10 million people, and caused >500 thousand deaths worldwide (1). As a result, the last few months has seen a rapid expansion of COVID-19 research (2–7) including the discovery of potential inhibitors and drugs against SARS-CoV-2 (8–12).

The SARS-CoV-2 genome contains ∼30 000 nucleotides that encode about 29 proteins such as spike protein, 3C-like protease (3CLpro, also called main protease, Mpro), Papain-like protease (PLpro), RNA-dependent RNA polymerase (RdRp, also named nsp12), and nucleocapsid (N) protein (9,13–15). These five proteins are all known to play important roles in the viral replication process in some way. For example, the surface spike glycoprotein is known to be critical for virus entry through binding to the major antigens of host receptors such as angiotensin-converting enzyme 2 (ACE2) (16,17). The 3CLpro is a key enzyme which digests pp1a and pp1ab, two proteins that yield functional viral proteins in the virus' life cycle (10,18). Blocking the activity of this enzyme would therefore suppress viral replication, making it an efficient therapeutic strategy. Likewise, PLpro is responsible for the cleavage of the viral polypeptide (19), and RdRp catalyzes viral RNA synthesis and plays an important role in the replication and transcription machinery of SARS-CoV-2 (20). Remdesivir, the potent inhibitor against SARS-CoV-2, is effective because of how it targeted the RdRp protein (20,21). N protein is an RNA-binding protein necessary for viral RNA transcription and replication (15). In addition to the viral proteins, SARS-CoV-2 uses human ACE2 for viral entry and human transmembrane serine protease family member II (TMPRSS2) for spike protein priming (16). We therefore hypothesize that these five SARS-CoV-2 proteins, spike, 3CLpro, PLpro, RdRp, N protein, and two host proteins, ACE2 and TMPRSS2, can be potential druggable targets.

Previously, researchers have targeted these viral proteins for drug discovery by using structure-assisted design, virtual and high-throughput screening (8,11). Similarly, these approaches can also be used for SARS-CoV-2 research (22). For example, Jin *et al.* used a computer-aided drug design and virtual screening to identify the mechanism-based inhibitor (N3) and the antineoplastic drug carmofur targeting Mpro (10,11). They further determined the crystal structures of the N3- and carmofur-Mpro complexes and confirmed the antiviral activities of N3 and carmofur using the plaque-reduction assays (10,11).

Drug repositioning is the process of finding new uses for existing approved drugs, and is believed to offer great benefits over *de novo* drug discovery, as well as enable rapid clinical trials and regulatory review for COVID-19 therapy (23). Here we develop the database, DockCoV2, by performing molecular docking analyses of seven proteins including spike, 3CLpro, PLpro, RdRp, N protein, ACE2 and TMPRSS2 with 2285 FDA-approved and 1478 NHI drugs. DockCoV2 also provides appropriate experimental information with literature support. Several databases focus on delivering repurposing drugs against SARS-CoV-2. For example, Excelra's COVID-19 Drug Repurposing Database includes 128 small molecules and biologics (https://www.excelra.com/covid-19-drug-repurposing-database/) and the OpenData collected the actionable data and validated methodologies (https://doi.org/10.1101/2020.06.04.135046). Additionally, Covid19_DB lists the curated data of clinical trials in Covid-19/2019-nCoV (http://www.redo-project.org/covid19db/). To our knowledge, no database provides a more up-to-date and comprehensive resource with drug-target docking results for repurposed drugs against SARS-CoV-2.

## DATA SOURCES

For drug repositioning, we selected FDA and Taiwan NHI (National Health Insurance Administration) as our list of approved drugs. FDA-approved drugs were retrieved from the ZINC15 database (24), and Taiwan NHI-approved drugs were downloaded from the website of NHI. Drug names were extracted from the top five ingredients of each product, and drug structures were obtained from the PubChem Compound database (25). Due to the limitation of the ligand preparation pipeline, we dropped some compounds with ions which could not be converted properly by the gen3d operation of OpenBabel (version 3.0.0) (26). Note that we also added some additional FDA-approved drugs which are not in the ZINC15 database. In total, 2285 FDA-approved drugs and 1478 Taiwan NHI-approved drugs were recruited as our candidates for virtual screening.

Seven target proteins were selected because of their involvement in viral entry and replication. The protein structures, including 3CLpro (PDB ID: 6LU7) (10), PLpro (PDB ID: 6WX4) (https://doi.org/10.1101/2020.04.29.068890), RdRp (PDB ID: 7BV2) (21), spike receptor-binding domain (RBD) (PDB ID: 6M0J) (27), N protein (PDB ID: 6M3M) (15) and ACE2 (PDB ID: 1R42) (28) were retrieved from Protein Data Bank. To ensure the availability of the binding sites of the proteins, we removed the compounds added for protein structure determination such as ligands, cofactors, and ions. For the spike protein, only the RBD was used for molecular docking and for 7BV2, only the nsp12 was used out of the nsp7–nsp8–nsp12 complex. The structure of human TMPRSS2 is currently not available in Protein Data Bank, so we used homology modeling on the sequence of TMPRSS2 from Uniprot (ID: O15393) (29) to build an approximate structure based on the template of the human serine protease hepsin (PDB ID: 5CE1) using SWISS-MODEL (30).

## DATABASE CONTENT

DockCov2 aims to speed up the process of finding potential drugs by providing a computational representation of molecular docking with the most commonly-used drug databases (31–35). Through our website, researchers can query the docking scores of candidate drugs with essential drug-related information from the database to identify which drugs could be potential drugs. We designed a virtual screening pipeline, and docked 2285 FDA approved drugs and 1478 Taiwan National Health Insurance (NHI) approved drugs with the seven main proteins of the SARS-CoV-2 infection mechanism (36–38). All of our docking results are saved in the formats of Protein Data Bank, Partial Charge (Q) and Atom Type (T) (PDBQT), and can be viewed either directly on the website through NGLView (39) or downloaded for further docked structure refinement.

**Figure 1.**
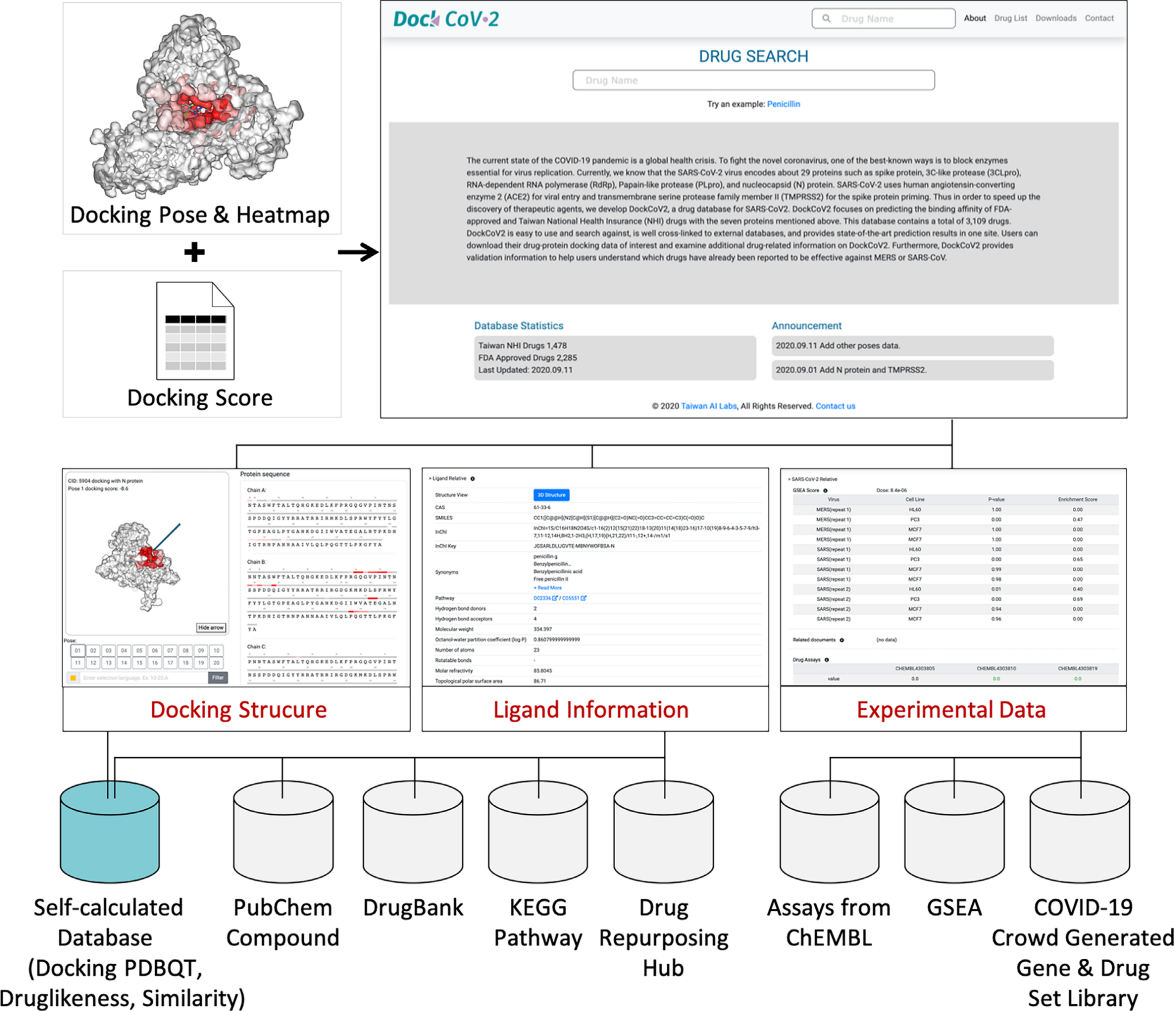
The overview of the database content. In addition to the docking scores, DockCoV2 designed a joint panel section to provide the following related information: docking structure, ligand information and experimental data.

The overview of the database is shown in Figure 1. In addition to the molecular docking score, the joint panel section has three tabs: docking structure, ligand information and experimental data. The docking structure panel visualizes the docking PDBQT from the self-calculated database, which also contains druglikeness information and compound similarity. The ligand information panel contains (a) structural information from PubChem Compound database (25); (b) drug information from DrugBank (33); (c) pathway information from Kyoto Encyclopedia of Genes and Genomes (KEGG) (40); (d) clinical-related information from Repurposing Hub (34); and (e) other chemical information. In the experimental data panel, we collected experimental and literary evidence from ChEMBL (35), a study of gene set enrichment analysis (GSEA) from Zhou *et al.* (41) and COVID-19 Crowd Generated Gene and Drug Set Library (https://amp.pharm.mssm.edu/covid19).

## DATA PROCESSING AND INTEGRATION

### Identifier conversion

We chose PubChem compound identifiers (CIDs) as the key identifier in DockCoV2 because it is unique and widely used and can be easily linked to other external databases. For drugs retrieved from NHI, drug names were translated into CIDs using whole source mappings provided by UniChem (31) and PubChem Identifier Exchange Service (https://pubchem.ncbi.nlm.nih.gov/idexchange/). Specifically, multiple CIDs may be returned by PubChem Identifier Exchange Service for each drug name. We canonically chose the first returned record in order to be consistent with the result by manual search via the graphical interface of PubChem. Once we have the PubChem CIDs of our compound library, simplified molecular-input line-entry system (SMILES) (42), international chemical identifier (InChI), hashed values of international chemical identifiers (InChIKey), and synonyms were fetched through the PubChem PUG API (43).

### Ligand information

For drug development, Lipinski's rule of five (44), Ghose filter (45), Veber filter (46), rapid elimination of swill (REOS) filter (47) are several available indicators to evaluate the druglikeness. Therefore, components of these rules were calculated by RDKit (http://www.rdkit.org). Moreover, fragment screening is able to find a sensible starting point for the evolution of a new molecule in modern drug discovery. Previously, a large crystallographic fragment screen (48) was performed against 3CLpro by the Diamond Light Source group. Seventy-eight hits were released to the public in March 2020. In DockCoV2, we performed the substructure matching of these fragments toward the drug library we used here. To let the user easily fetch structurally similar compounds, similarity search (49) strategies were also adopted. Similarity search within the DockCoV2 and toward whole PubChem compound databases are both supported. To generate similarity values between any two compounds in DockCoV2, a 512-bit fingerprint of each compound is calculated using the Morgan algorithm (50). Each bit represents the presence or absence of a particular structural feature. From here, similarity values between any compound pairs are represented by the Dice coefficient. In the current version of DockCoV2, the rule of five, substructure matching, and the calculation of similarity values were all done by using the RDKit 2020.03.2 release.

Pathway information can serve as the basis of hypothesis in drug discovery. Therefore, two databases of KEGG (namely, KEGG Drug and KEGG Compound) were integrated into DockCoV2. PubChem CIDs were converted into DrugBank IDs first, then further converted into KEGG Drug IDs and KEGG Compound IDs. Alternatively, PubChem CIDs were directly converted to KEGG Drug IDs and KEGG Compound IDs by PubChem Identifier Exchange Service. Clinically related information including approval information, clinical phase, mechanism of action (MoA), target, disease area, and indication were also retrieved from the metadata file from the Drug Repurposing Hub.

### Experimental data

To provide useful validation information for drug repurposing and development, chemical information was aggregated either from external databases or directly calculated from structures. Three biological assays CHEMBL4303805, CHEMBL4303810, CHEMBL4303819 were fetched from ChEMBL (35). All of these three assays are functional assays. For CHEMBL4303805, Antiviral activities were determined as inhibition of SARS-CoV-2 induced cytotoxicity of Caco-2 cells at 10 uM after 48 h by high content imaging. Ratios of inhibition of each compound were reported. For CHEMBL4303810, Antiviral activities against SARS-CoV-2 (USA-WA1/2020 strain) were measured by imaging in HRCE cells at MOI 0.4 after 96 h. Hit scores were reported from 0 to 1 for on-disease versus off-disease activity: scores >0.6 are considered hits. For CHEMBL4303819, Inhibition of cell viability relative to arbidol control (inhibition index) was measured by fluorescence (OD590nm) in Vero E6 cells, which were infected with SARS-CoV-2 (strain BavPat1) at MOI 0.002 after 72 h. Inhibition index with a value over 1 indicates higher activity. ChEMBL IDs were translated into CIDs using whole source mappings provided by UniChem (31) and PubChem Identifier Exchange Service as well.

Gene Set Enrichment Analysis (GSEA) is a method that determines whether an a priori defined set of genes show statistically significant expression differences between two biological states (51). Here, three NCBI Gene Expression Omnibus (GEO) data, GSE1739, GSE33267 and GSE122876 were used to identify sets of differentially expressed genes post SARS or MERS infection. Gene expression profiles of drug treatment were retrieved from the Connectivity Map (CMAP) database. The GSEA enrichment scores indicate the potential effectiveness of candidate drugs to reverse the gene expression signature of HCoV infection. For detailed information, please see Zhou *et al.* (41). Literature was also fetched from COVID 19 Coward Generated Gene and Drug Set Library to assist the user to validate the hypothesis of an interaction between a drug and its target. Only compounds that were reported in experiments were collected.

## VIRTUAL SCREENING

**Figure 2.**
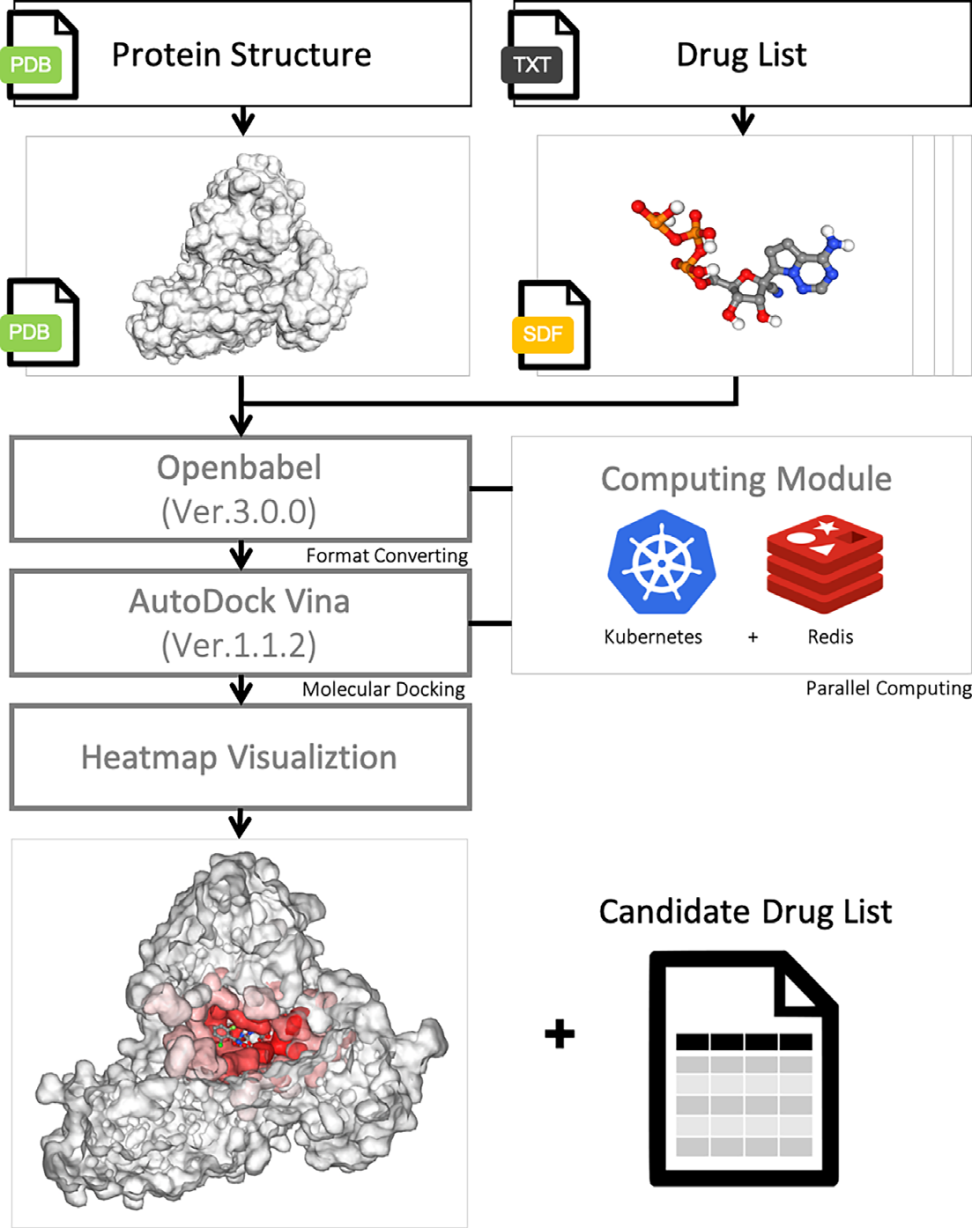
The virtual screening pipeline. We used AutoDock Vina as the core docking utility to reconstruct the virtual screening pipeline. In addition, the Kubernetes server and Redis data store were adopted for parallel computing. After finishing docking, the top 20 docking poses were taken to build a protein heatmap for visualization.

There are many softwares that exist for virtual screening such as VSDK (52), AUDocker (53), DockoMatic (54), PyRx (55), etc. However, the efficiency of these tools may not be applicable on large drug banks such as the complete FDA-approved drug list. Therefore, we used AutoDock Vina (version 1.1.2) (56) as the core docking utility to reconstruct our virtual screening pipeline, and ran it on a Kubernetes server with parallel computing (Figure 2). Kubernetes is an engine server, which can automatically deploy, scale and manage the containerized tasks to clusters. In practice, we uploaded the FDA-approved and Taiwan NHI-approved drug lists to Redis data storage, and generated 128 Kuberenetes pods in parallel. Each pod continuously takes up a new docking task of a drug until the Redis drug list is completed. Based on our implementation, the virtual screening for our drug list only costs 3 days per protein.

**Figure 3.**
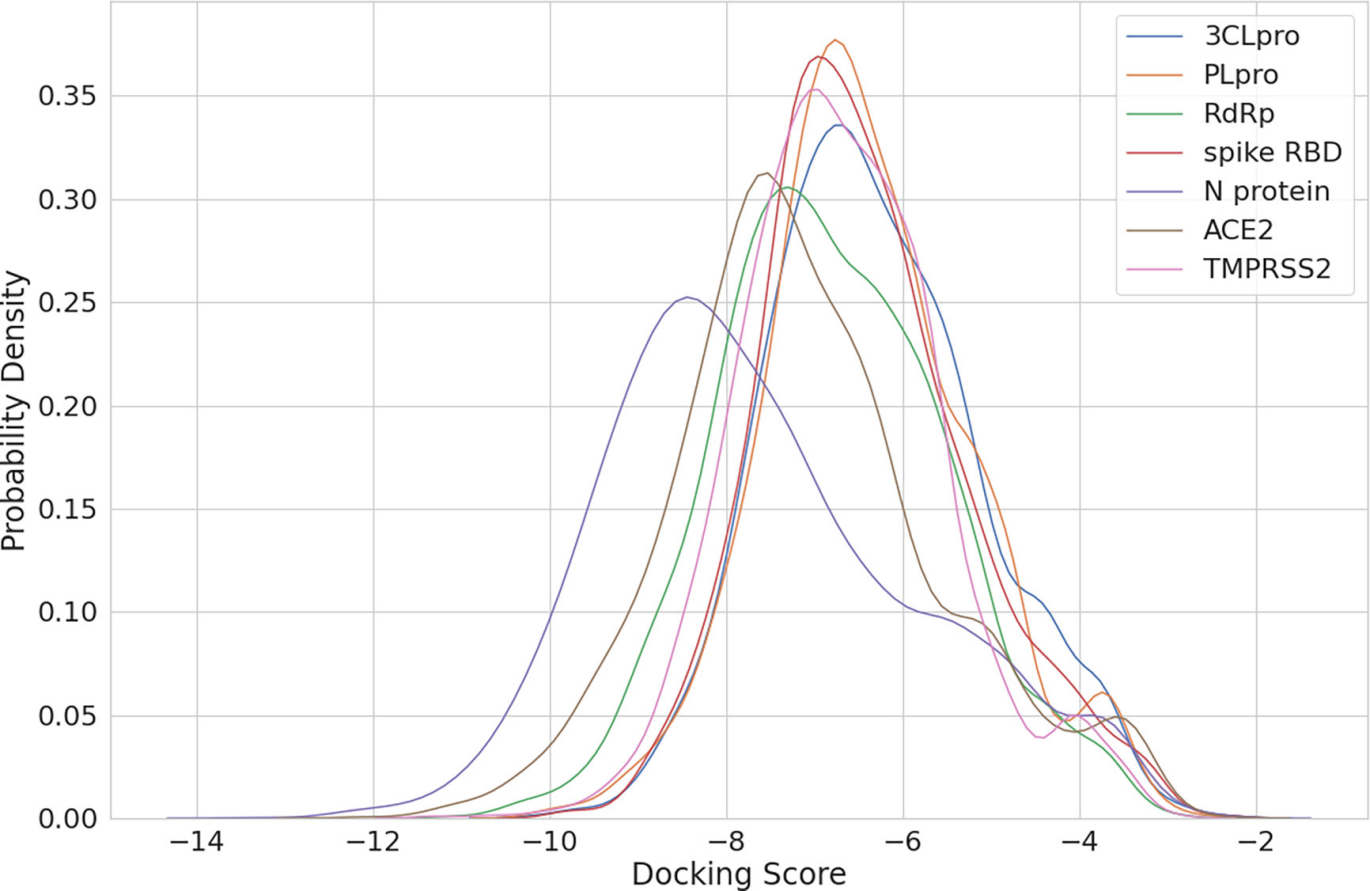
The docking score distribution of each protein. The x-axis is the minimum docking score of each protein–ligand pair, and the y-axis is the probability density estimated by kernel density estimation.

To simulate binding affinity between protein and ligands, each of the drug's 3D structure was obtained from the PubChem database in structure-data file (SDF) format. The gen3d operation of OpenBabel (version 3.0.0) (26) was used for energy minimization. This operation iterated 500 cycles of performing the geometry optimization with the MMFF94 force field and conducting the weighted rotor conformational search, in order to generate a likely global minimum energy conformer in MOL2 file format. Since AutoDock Vina only takes the PDBQT format as input, we used AutoDockTool 1.5.7 (http://autodock.scripps.edu/resources/adt) to convert the file format from MOL2 to PDBQT with default parameter settings excluding a parameter -A, which would add hydrogens to structure only if there are none already. After that, we applied rigid-body docking on these converted files using AutoDock Vina. In order to consider all of the potential docking poses, the entire protein is taken as the search space for blind search. We noted that the number of runs of the docking simulation should be adjusted accordingly by considering the variety of protein sizes. In AutoDock Vina, the number of runs is set by the exhaustiveness parameter, and the default setting of exhaustiveness is set at eight for a search space below 30 × 30 × 30 Å (56). We proportionally scaled the exhaustiveness depending on the size of the protein by a factor of 2. For example, if the size of a protein is 60 × 60 × 60 Å, the exhaustiveness would be 8 × (60/30) × (60 /30) × (60 /30) × 2 = 128. AutoDock Vina reported multiple docking scores for each run, and the top score was selected as the final result shown on the website. The distribution of the final scores of each protein is shown in Figure 3. By comparing the scores between systems (different proteins), there is no bias of docking scores toward any protein we are interested in. By examining the intra-system scores, we observed that for each protein if there is a group or few ligands that are with particularly good docking scores.

**Figure 4.**
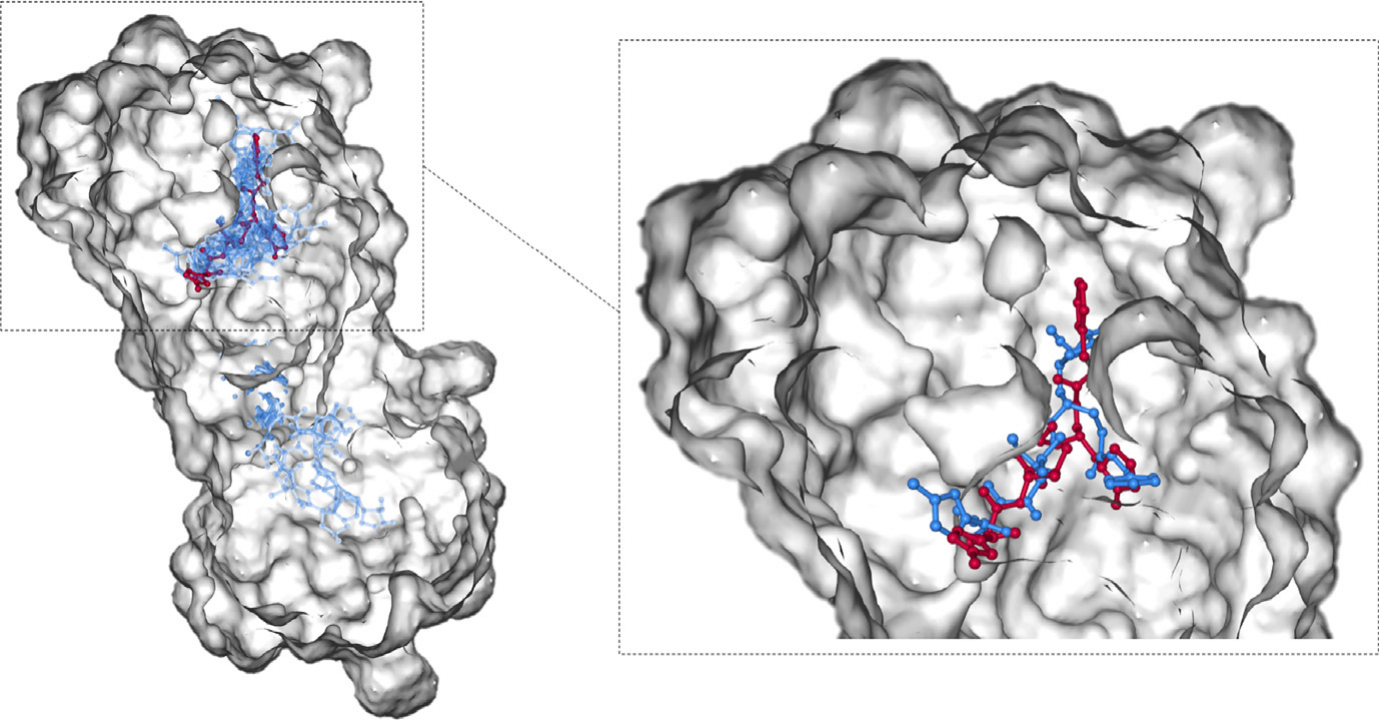
A demonstration of the bound structure of 3CLpro (PDB ID: 6LU7, where the ligand was colored in red) with molecular docking. The left-hand side shows the results of the top 20 docking poses (colored in blue). Eight out of 20 docking poses are located in the binding pocket of the protein. The right-hand side zooms in the binding pocket and shows that the top docking pose (colored in blue) is close to the bound ligand (colored in red).

After finishing docking, AutoDock Vina will generate multiple docking poses for each ligand–protein pair. For the sake of a straightforward representation of the docking results, the top 20 docking poses from AutoDock Vina were taken to build a protein heatmap. For each pose, a sphere centered at the centroid of ligand with a radius of 5 Å is highlighted, and the frequency of residues inside the sphere is incremented. The protein heatmap and the ligand docking position with the best affinity score are shown on the main page of each entry (a ligand–protein pair) in DockCoV2, where the red scale of the protein heatmap denotes the frequency of residues over twenty trials. Figure 4 is a demonstration of the structure of 3CLpro (PDB ID: 6LU7) (10) with molecular docking. In order to validate our virtual screening pipeline, we separated the original structure into a ligand and a receptor, and docked them with the proposed pipeline. The left-hand side of Figure 4 shows that eight out of the top 20 docking poses (colored in blue) are located in the binding pocket of the protein. This figure also demonstrates that users can explore the binding pocket through the hot spot of our heatmap. The right-hand side zooms in the binding pocket and shows that the top docking pose (colored in blue) of our pipeline is close to the ligand (colored in red) in the crystal structure. For reproducing our results or repurposing the proposed pipeline for other drugs or proteins, we released our scripts and the configuration file for docking procedures on GitHub (https://github.com/ailabstw/DockCoV2).

## INTERFACE

In order to provide an efficient searching interface for users, we built a website with a search engine based on Django 3.0 (https://djangoproject.com) and Bootstrap 4.3.1 (https://getbootstrap.com/). The search bar, which can be found either in the index page or on navbar, allows the user to input the drug name, PubChem CID or synonym to explore the docking score with all proteins. The details of each ligand are shown after choosing a specific ligand–protein pair, including docking structure, ligand information and experimental data. PDBQT files of ligand and protein from AutoDock Vina are also provided on the main page of each ligand-protein pair. In the docking structure panel, both protein heatmap and sequences are displayed on the right-side of the detail page. Protein heatmap is interactively demonstrated with the NGL viewer tool, which is a WebGL-based molecular viewer (57). To easily visualize the targeted binding sites on the proteins, not only basic operations on protein heatmap, but also selecting or limiting atoms/residues through the selection language is available on the docking structure panel. The ligand information panel is divided into ligand relative, clinical relative, druglikeness, other relative info and drug similarity. Ligand relative part consists of basic compound Information, including 3D structure view, CAS number, SMILES, InChI, InChI key, synonyms and pathway from KEGG Drug and KEGG Compound databases. Clinical Relative consists of approval information, which denotes whether or not this ligand has been approved by the Taiwan NHI or FDA, as well as other corresponding data from Drug Repurposing Hub, including clinical phase, mechanism of action (MoA), target, disease area, and indication. Results and components of Lipinski's rule of five, Ghose filter, Veber filter and REOS filter are shown in druglikeness section. External links to PubChem and Drugbank are also provided in the other relative info section. SARS-CoV-2 relative, related documents and drug assays are included in experimental data.

## CONCLUSION

Drug repositioning, the exploration of repurposing existing drugs that have already passed the safety screening for new indications, is a relatively quick therapeutic solution for emerging infectious diseases like SARS-CoV-2. For the purpose of drug repositioning, molecular docking is a well-known virtual screening method to evaluate a great amount of compounds automatically. Based on the urgent need to find effective drugs, we established DockCoV2, the first virtual screening database for SARS-CoV-2. DockCoV2 focuses on predicting 2285 FDA-approved and 1478 Taiwan NHI-approved drugs targeting five proteins relating to the mechanism of viral entry and replication. Those docked structures could also be further utilized and get more accurate binding poses and the correct ranking of the binding affinity through methods such as molecular mechanics Poisson-Boltzman surface area (MM/PBSA) and molecular mechanics generalized Boltzman surface area (MM/GBSA) calculations (22,58). In addition, DockCoV2 also provides experimental data, including biological assays, pathway information, and gene set enrichment analysis recruited from other validated databases. Future versions will include the prediction of binding affinity with other potentially druggable targets against SARS-CoV-2 and the reported experimental assays and high-throughput screening results to support our prediction.

## References

[1] Dong E., Du H., Gardner L. An interactive web-based dashboard to track COVID-19 in real time. Lancet Infect. Dis. 2020; 20:533–534. 32087114 10.1016/S1473-3099(20)30120-1 PMC7159018

[2] Bost P., Giladi A., Liu Y., Bendjelal Y., Xu G., David E., Blecher-Gonen R., Cohen M., Medaglia C., Li H. et al. Host-viral infection maps reveal signatures of severe COVID-19 patients. Cell. 2020; 181:1475–1488. 32479746 10.1016/j.cell.2020.05.006 PMC7205692

[3] Cao Y., Su B., Guo X., Sun W., Deng Y., Bao L., Zhu Q., Zhang X., Zheng Y., Geng C. et al. Potent neutralizing antibodies against SARS-CoV-2 identified by high-throughput single-cell sequencing of convalescent patients’ B cells. Cell. 2020; 182:73–84. 32425270 10.1016/j.cell.2020.05.025 PMC7231725

[4] Grifoni A., Weiskopf D., Ramirez S.I., Mateus J., Dan J.M., Moderbacher C.R., Rawlings S.A., Sutherland A., Premkumar L., Jadi R.S. et al. Targets of T cell responses to SARS-CoV-2 coronavirus in humans with COVID-19 disease and unexposed individuals. Cell. 2020; 181:1489–1501. 32473127 10.1016/j.cell.2020.05.015 PMC7237901

[5] Hou Y.J., Okuda K., Edwards C.E., Martinez D.R., Asakura T., Dinnon K.H. 3rd, Kato T., Lee R.E., Yount B.L., Mascenik T.M. et al. SARS-CoV-2 reverse genetics reveals a variable infection gradient in the respiratory tract. Cell. 2020; 182:429–446. 32526206 10.1016/j.cell.2020.05.042 PMC7250779

[6] Park A., Iwasaki A. Type I and Type III interferons - induction, signaling, evasion, and application to combat COVID-19. Cell Host Microbe. 2020; 27:870–878. 32464097 10.1016/j.chom.2020.05.008 PMC7255347

[7] Wilk A.J., Rustagi A., Zhao N.Q., Roque J., Martínez-Colón G.J., McKechnie J.L., Ivison G.T., Ranganath T., Vergara R., Hollis T. et al. A single-cell atlas of the peripheral immune response in patients with severe COVID-19. Nat. Med. 2020; 26:1070–1076. 32514174 10.1038/s41591-020-0944-y PMC7382903

[8] Dai W., Zhang B., Jiang X.-M., Su H., Li J., Zhao Y., Xie X., Jin Z., Peng J., Liu F. et al. Structure-based design of antiviral drug candidates targeting the SARS-CoV-2 main protease. Science. 2020; 368:1331–1335. 32321856 10.1126/science.abb4489 PMC7179937

[9] Gordon D.E., Jang G.M., Bouhaddou M., Xu J., Obernier K., White K.M., O’Meara M.J., Rezelj V.V., Guo J.Z., Swaney D.L. et al. A SARS-CoV-2 protein interaction map reveals targets for drug repurposing. Nature. 2020; 583:459–468. 32353859 10.1038/s41586-020-2286-9 PMC7431030

[10] Jin Z., Du X., Xu Y., Deng Y., Liu M., Zhao Y., Zhang B., Li X., Zhang L., Peng C. et al. Structure of M from SARS-CoV-2 and discovery of its inhibitors. Nature. 2020; 582:289–293. 32272481 10.1038/s41586-020-2223-y

[11] Jin Z., Zhao Y., Sun Y., Zhang B., Wang H., Wu Y., Zhu Y., Zhu C., Hu T., Du X. et al. Structural basis for the inhibition of SARS-CoV-2 main protease by antineoplastic drug carmofur. Nat. Struct. Mol. Biol. 2020; 27:529–532. 32382072 10.1038/s41594-020-0440-6

[12] Wang Q., Wu J., Wang H., Gao Y., Liu Q., Mu A., Ji W., Yan L., Zhu Y., Zhu C. et al. Structural basis for RNA replication by the SARS-CoV-2 polymerase. Cell. 2020; 182:417–428. 32526208 10.1016/j.cell.2020.05.034 PMC7242921

[13] Wu F., Zhao S., Yu B., Chen Y.-M., Wang W., Song Z.-G., Hu Y., Tao Z.-W., Tian J.-H., Pei Y.-Y. et al. A new coronavirus associated with human respiratory disease in China. Nature. 2020; 579:265–269. 32015508 10.1038/s41586-020-2008-3 PMC7094943

[14] Zhou P., Yang X.-L., Wang X.-G., Hu B., Zhang L., Zhang W., Si H.-R., Zhu Y., Li B., Huang C.-L. et al. A pneumonia outbreak associated with a new coronavirus of probable bat origin. Nature. 2020; 579:270–273. 32015507 10.1038/s41586-020-2012-7 PMC7095418

[15] Kang S., Yang M., Hong Z., Zhang L., Huang Z., Chen X., He S., Zhou Z., Zhou Z., Chen Q. et al. Crystal structure of SARS-CoV-2 nucleocapsid protein RNA binding domain reveals potential unique drug targeting sites. Acta Pharm. Sin. B. 2020; 10:1228–1238. 32363136 10.1016/j.apsb.2020.04.009 PMC7194921

[16] Hoffmann M., Kleine-Weber H., Schroeder S., Krüger N., Herrler T., Erichsen S., Schiergens T.S., Herrler G., Wu N.-H., Nitsche A. et al. SARS-CoV-2 cell entry depends on ACE2 and TMPRSS2 and is blocked by a clinically proven protease inhibitor. Cell. 2020; 181:271–280. 32142651 10.1016/j.cell.2020.02.052 PMC7102627

[17] Yuan M., Wu N.C., Zhu X., Lee C.-C.D., So R.T.Y., Lv H., Mok C.K.P., Wilson I.A. A highly conserved cryptic epitope in the receptor binding domains of SARS-CoV-2 and SARS-CoV. Science. 2020; 368:630–633. 32245784 10.1126/science.abb7269 PMC7164391

[18] Zhang L., Lin D., Sun X., Curth U., Drosten C., Sauerhering L., Becker S., Rox K., Hilgenfeld R. Crystal structure of SARS-CoV-2 main protease provides a basis for design of improved α-ketoamide inhibitors. Science. 2020; 368:409–412. 32198291 10.1126/science.abb3405 PMC7164518

[19] Freitas B.T., Durie I.A., Murray J., Longo J.E., Miller H.C., Crich D., Hogan R.J., Tripp R.A., Pegan S.D. Characterization and noncovalent inhibition of the deubiquitinase and deISGylase activity of SARS-CoV-2 papain-like protease. ACS Infect Dis. 2020; 6:2099–2109. 32428392 10.1021/acsinfecdis.0c00168

[20] Gao Y., Yan L., Huang Y., Liu F., Zhao Y., Cao L., Wang T., Sun Q., Ming Z., Zhang L. et al. Structure of the RNA-dependent RNA polymerase from COVID-19 virus. Science. 2020; 368:779–782. 32277040 10.1126/science.abb7498 PMC7164392

[21] Yin W., Mao C., Luan X., Shen D.-D., Shen Q., Su H., Wang X., Zhou F., Zhao W., Gao M. et al. Structural basis for inhibition of the RNA-dependent RNA polymerase from SARS-CoV-2 by remdesivir. Science. 2020; 368:1499–1504. 32358203 10.1126/science.abc1560 PMC7199908

[22] Panda P.K., Arul M.N., Patel P., Verma S.K., Luo W., Rubahn H.-G., Mishra Y.K., Suar M., Ahuja R. Structure-based drug designing and immunoinformatics approach for SARS-CoV-2. Sci. Adv. 2020; 6:eabb8097. 32691011 10.1126/sciadv.abb8097 PMC7319274

[23] Guy R.K., Kiplin Guy R., DiPaola R.S., Romanelli F., Dutch R.E. Rapid repurposing of drugs for COVID-19. Science. 2020; 368:829–830. 32385101 10.1126/science.abb9332

[24] Sterling T., Irwin J.J. ZINC 15 – ligand discovery for everyone. J. Chem. Inf. Model. 2015; 55:2324–2337. 26479676 10.1021/acs.jcim.5b00559 PMC4658288

[25] Kim S., Thiessen P.A., Bolton E.E., Chen J., Fu G., Gindulyte A., Han L., He J., He S., Shoemaker B.A. et al. PubChem substance and compound databases. Nucleic Acids Res. 2016; 44:D1202–D1213. 26400175 10.1093/nar/gkv951 PMC4702940

[26] O’Boyle N.M., Banck M., James C.A., Morley C., Vandermeersch T., Hutchison G.R. Open Babel: an open chemical toolbox. J. Cheminform. 2011; 3:33. 21982300 10.1186/1758-2946-3-33 PMC3198950

[27] Lan J., Ge J., Yu J., Shan S., Zhou H., Fan S., Zhang Q., Shi X., Wang Q., Zhang L. et al. Structure of the SARS-CoV-2 spike receptor-binding domain bound to the ACE2 receptor. Nature. 2020; 581:215–220. 32225176 10.1038/s41586-020-2180-5

[28] Towler P., Staker B., Prasad S.G., Menon S., Tang J., Parsons T., Ryan D., Fisher M., Williams D., Dales N.A. et al. ACE2 X-ray structures reveal a large hinge-bending motion important for inhibitor binding and catalysis. J. Biol. Chem. 2004; 279:17996–18007. 14754895 10.1074/jbc.M311191200 PMC7980034

[29] UniProt Consortium UniProt: a worldwide hub of protein knowledge. Nucleic Acids Res. 2019; 47:D506–D515. 30395287 10.1093/nar/gky1049 PMC6323992

[30] Waterhouse A., Bertoni M., Bienert S., Studer G., Tauriello G., Gumienny R., Heer F.T., de Beer T.A.P., Rempfer C., Bordoli L. et al. SWISS-MODEL: homology modelling of protein structures and complexes. Nucleic Acids Res. 2018; 46:W296–W303. 29788355 10.1093/nar/gky427 PMC6030848

[31] Chambers J., Davies M., Gaulton A., Hersey A., Velankar S., Petryszak R., Hastings J., Bellis L., McGlinchey S., Overington J.P. UniChem: a unified chemical structure cross-referencing and identifier tracking system. J. Cheminform. 2013; 5:3. 23317286 10.1186/1758-2946-5-3 PMC3616875

[32] Kim S., Chen J., Cheng T., Gindulyte A., He J., He S., Li Q., Shoemaker B.A., Thiessen P.A., Yu B. et al. PubChem 2019 update: improved access to chemical data. Nucleic Acids Res. 2019; 47:D1102–D1109. 30371825 10.1093/nar/gky1033 PMC6324075

[33] Wishart D.S., Feunang Y.D., Guo A.C., Lo E.J., Marcu A., Grant J.R., Sajed T., Johnson D., Li C., Sayeeda Z. et al. DrugBank 5.0: a major update to the DrugBank database for 2018. Nucleic Acids Res. 2018; 46:D1074–D1082. 29126136 10.1093/nar/gkx1037 PMC5753335

[34] Corsello S.M., Bittker J.A., Liu Z., Gould J., McCarren P., Hirschman J.E., Johnston S.E., Vrcic A., Wong B., Khan M. et al. The Drug Repurposing Hub: a next-generation drug library and information resource. Nat. Med. 2017; 23:405–408. 28388612 10.1038/nm.4306 PMC5568558

[35] Mendez D., Gaulton A., Bento A.P., Chambers J., De Veij M., Félix E., Magariños M.P., Mosquera J.F., Mutowo P., Nowotka M. et al. ChEMBL: towards direct deposition of bioassay data. Nucleic Acids Res. 2019; 47:D930–D940. 30398643 10.1093/nar/gky1075 PMC6323927

[36] Li G., De Clercq E. Therapeutic options for the 2019 novel coronavirus (2019-nCoV). Nat. Rev. Drug Discov. 2020; 19:149–150. 32127666 10.1038/d41573-020-00016-0

[37] Zumla A., Chan J.F.W., Azhar E.I., Hui D.S.C., Yuen K.-Y. Coronaviruses - drug discovery and therapeutic options. Nat. Rev. Drug Discov. 2016; 15:327–347. 26868298 10.1038/nrd.2015.37 PMC7097181

[38] Wang Q., Zhang Y., Wu L., Niu S., Song C., Zhang Z., Lu G., Qiao C., Hu Y., Yuen K.-Y. et al. Structural and functional basis of SARS-CoV-2 entry by using human ACE2. Cell. 2020; 181:894–904. 32275855 10.1016/j.cell.2020.03.045 PMC7144619

[39] Nguyen H., Case D.A., Rose A.S. NGLview–interactive molecular graphics for Jupyter notebooks. Bioinformatics. 2018; 34:1241–1242. 29236954 10.1093/bioinformatics/btx789 PMC6031024

[40] Kanehisa M. KEGG: Kyoto encyclopedia of genes and genomes. Nucleic Acids Res. 2000; 28:27–30. 10592173 10.1093/nar/28.1.27 PMC102409

[41] Zhou Y., Hou Y., Shen J., Huang Y., Martin W., Cheng F. Network-based drug repurposing for novel coronavirus 2019-nCoV/SARS-CoV-2. Cell Discov. 2020; 6:14. 32194980 10.1038/s41421-020-0153-3 PMC7073332

[42] Weininger D. SMILES, a chemical language and information system. 1. Introduction to methodology and encoding rules. J. Chem. Inf. Model. 1988; 28:31–36.

[43] Kim S., Thiessen P.A., Cheng T., Zhang J., Gindulyte A., Bolton E.E. PUG-View: programmatic access to chemical annotations integrated in PubChem. J. Cheminform. 2019; 11:56. 31399858 10.1186/s13321-019-0375-2 PMC6688265

[44] Lipinski C.A., Lombardo F., Dominy B.W., Feeney P.J. Experimental and computational approaches to estimate solubility and permeability in drug discovery and development settings. Adv. Drug Deliv. Rev. 2001; 46:3–26. 11259830 10.1016/s0169-409x(00)00129-0

[45] Ghose A.K., Viswanadhan V.N., Wendoloski J.J. A knowledge-based approach in designing combinatorial or medicinal chemistry libraries for drug discovery. 1. A qualitative and quantitative characterization of known drug databases. J. Comb. Chem. 1999; 1:55–68. 10746014 10.1021/cc9800071

[46] Veber D.F., Johnson S.R., Cheng H.-Y., Smith B.R., Ward K.W., Kopple K.D. Molecular properties that influence the oral bioavailability of drug candidates. J. Med. Chem. 2002; 45:2615–2623. 12036371 10.1021/jm020017n

[47] Walters W.P., Namchuk M. Designing screens: how to make your hits a hit. Nat. Rev. Drug Discov. 2003; 2:259–266. 12669025 10.1038/nrd1063

[48] Hubbard R.E. Hubbard R.E. Fragment Screening: An Introduction. Structure-Based Drug Discovery. 2006; Royal Society of Chemistry 142–172.

[49] Willett P., Barnard J.M., Downs G.M. Chemical similarity searching. J. Chem. Inf. Comput. Sci. 1998; 38:983–996.

[50] Rogers D., Hahn M. Extended-connectivity fingerprints. J. Chem. Inf. Model. 2010; 50:742–754. 20426451 10.1021/ci100050t

[51] Subramanian A., Tamayo P., Mootha V.K., Mukherjee S., Ebert B.L., Gillette M.A., Paulovich A., Pomeroy S.L., Golub T.R., Lander E.S. et al. Gene set enrichment analysis: a knowledge-based approach for interpreting genome-wide expression profiles. Proc. Natl. Acad. Sci. USA. 2005; 102:15545–15550. 16199517 10.1073/pnas.0506580102 PMC1239896

[52] Baba N., Akaho E. VSDK: virtual screening of small molecules using AutoDock Vina on Windows platform. Bioinformation. 2011; 6:387–388. 21976864 10.6026/97320630006387 PMC3181425

[53] Sandeep G., Nagasree K.P., Hanisha M., Kumar M.M.K. AUDocker LE: a GUI for virtual screening with AUTODOCK Vina. BMC Res. Notes. 2011; 4:445. 22026969 10.1186/1756-0500-4-445 PMC3214202

[54] Bullock C., Cornia N., Jacob R., Remm A., Peavey T., Weekes K., Mallory C., Oxford J.T., McDougal O.M., Andersen T.L. DockoMatic 2.0: high throughput inverse virtual screening and homology modeling. J. Chem. Inf. Model. 2013; 53:2161–2170. 23808933 10.1021/ci400047w PMC3916141

[55] Dallakyan S., Olson A.J. Small-molecule library screening by docking with PyRx. Methods Mol. Biol. 2015; 1263:243–250. 25618350 10.1007/978-1-4939-2269-7_19

[56] Trott O., Olson A.J. AutoDock Vina: improving the speed and accuracy of docking with a new scoring function, efficient optimization, and multithreading. J. Comput. Chem. 2010; 31:455–461. 19499576 10.1002/jcc.21334 PMC3041641

[57] Rose A.S., Bradley A.R., Valasatava Y., Duarte J.M., Prlic A., Rose P.W. NGL viewer: web-based molecular graphics for large complexes. Bioinformatics. 2018; 34:3755–3758. 29850778 10.1093/bioinformatics/bty419 PMC6198858

[58] Wang E., Sun H., Wang J., Wang Z., Liu H., Zhang J.Z.H., Hou T. End-Point binding free energy calculation with MM/PBSA and MM/GBSA: strategies and applications in drug design. Chem. Rev. 2019; 119:9478–9508. 31244000 10.1021/acs.chemrev.9b00055

